# Effects of Aberrant miR-384-5p Expression on Learning and Memory in a Rat Model of Attention Deficit Hyperactivity Disorder

**DOI:** 10.3389/fneur.2019.01414

**Published:** 2020-02-11

**Authors:** Qu Xu, Jiaxin Ou, Qingyu Zhang, Ranran Tang, Jing Wang, Qin Hong, Xirong Guo, Meiling Tong, Lei Yang, Xia Chi

**Affiliations:** ^1^Department of Child Health Care, Women's Hospital of Nanjing Medical University (Nanjing Maternity and Child Health Care Hospital), Nanjing, China; ^2^Department of Pediatrics, First People's Hospital of Foshan, Affiliated Foshan Hospital of Sun Yat-sen University, Foshan, China; ^3^Jiangsu Key Laboratory of Pediatrics, Institute of Pediatrics, Nanjing Medical University, Nanjing, China; ^4^Tongren Hospital, Shanghai Jiao Tong University School of Medicine, Shanghai, China

**Keywords:** dopamine receptor D1, attention deficit hyperactivity disorder, learning and memory, cognition, microRNA, prefrontal cortex

## Abstract

Attention deficit hyperactivity disorder (ADHD) is a common neuropsychiatric disorder characterized by inattention, hyperactivity, and impulsivity. It may be accompanied by learning difficulties and working memory deficits. Few studies have examined the role of miRNAs in cognitive dysfunction in ADHD. This study investigated the effects of aberrant miR-384-5p expression on learning and memory in a widely used ADHD rat model. Lentiviral vectors were injected into the lateral ventricles of the rats to increase or decrease miR-384-5p level. To determine whether aberrant miR-384-5p expression affects learning and memory, spontaneous activity and cognitive function were assessed with the open field and Morris water maze tests. In the place navigation experiment of the Morris water maze test, time, and total swimming distance to reach the platform decreased compared to the control group when miR-384-5p was overexpressed, whereas down-regulation of miR-384-5p had the opposite effect. There were no obvious changes in brain tissue morphology following miR-384-5p overexpression or inhibition; however, dopamine (DA) receptor D1 (DRD1) level has decreased and increased, respectively, in the prefrontal cortex (PFC). The luciferase activity of the wild-type DRD1 group has decreased in luciferase reporter assay. Cyclic AMP response element-binding protein (CREB) phosphorylation has increased, and DA transporter (DAT) level has decreased in the PFC of spontaneously hypertensive rats (SHR) by miR-384-5p overexpression. On the other hand, miR-384-5p suppression increased DRD1 and decreased DAT and CREB protein levels relative to control rats. These findings suggest that miR-384-5p may play a critical role in learning and memory impairment in ADHD.

## Introduction

Attention deficit hyperactivity disorder (ADHD) is a common neuropsychiatric disorder that begins in childhood (1) and affects around 5% of children and adolescents (2). The main manifestations are inattention, hyperactivity, and emotional impulsivity (3). Learning disorders and memory (especially working memory) deficits may co-exist with ADHD. ADHD can lead to behavioral and social problems (4, 5) and may represent a burden to the individual, family, and society (6). The etiology and pathogenesis of ADHD remain poorly understood (7, 8).

The dopaminergic neurotransmitter system plays a critical role in ADHD (9). Dopamine (DA) is an endogenous neurotransmitter that acts through DA receptors (DR) of the D1-like (DRD1) and D2-like (DRD2) families. Methylphenidate, used for ADHD treatment, acts by blocking the DA transporter (DAT) (10). Cyclic AMP response element binding protein (CREB) has been linked to long-term synaptic plasticity and memory (11), which have been proposed to be related to ADHD.

MicroRNAs (miRNA) are non-coding small (19- to 24-nucleotide) RNAs that regulate gene expression post-transcriptionally by inhibiting mRNA translation or inducing mRNA degradation, although some miRNAs have a positive regulatory function. In the brain, miRNAs modulate cellular functions and participate in cell fate specification, neuronal survival, neurite projection, and synaptic plasticity (12). Around 70% of all known miRNAs are expressed in the brain, and some have been linked to neuropsychiatric disorders such as schizophrenia, bipolar disorder (13, 14), Tourette syndrome (15), and autism spectrum disorders (16–18). Recent evidence has suggested a role for miRNAs in ADHD (7, 19), implying that they could serve as therapeutic targets in ADHD treatment (20). For example, the cognitive behavior of rats is regulated by the miRNA miR-124 or miR-9 (21), and miR-335-5p was found to modulate spatial memory and hippocampal synaptic plasticity (22). However, there are few studies on the role of miRNAs in learning and memory, especially in the context of ADHD.

The miR-384-5p is highly expressed in the rat hippocampus (Hip) (23) and is essential for the maintenance of long-term potentiation (24). However, its role in learning and memory impairment in ADHD has not yet been reported. To address this issue, the present study investigated the function and mechanism of action of miR-384-5p in spontaneously hypertensive (SHR) rats, which is a frequently used animal model of ADHD.

## Experimental Procedures

### Animals

**Scheme 1:**
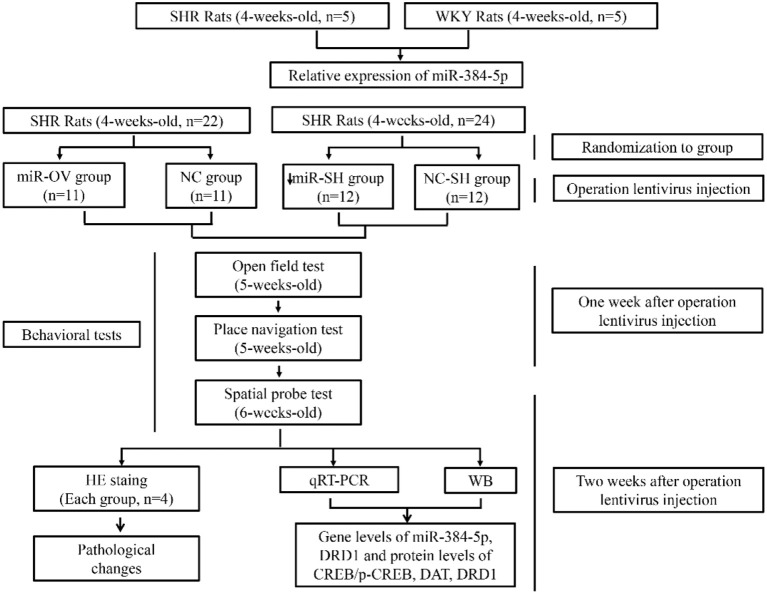
Schematic illustration of the experimental protocol. Shown are the sequence of experiments, age of rats for the behavioral tests, groups, and number of animals used.

Male SHR and Wistar Kyoto (WKY) rats (4 weeks old) were obtained from supplier Charles River, Beijing Vital River Laboratory Animal Technology Co. (Beijing, China), and were maintained in a controlled environment at 23 ± 1°C and a relative humidity of 50 ± 5% on a 12/12-h light/dark cycle. All animals had free access to food and water (Scheme 1). The research protocol was approved by the Animal Ethics Committee of Nanjing Medical University (approval no. IACUC-1711004).

### Stereotactic Injection of Lentivirus

The rats were anesthetized with 10% chloral hydrate (0.35 ml/100 g) by intraperitoneal injection. The depth of anesthesia was determined by monitoring reflexes, heart rate, skin color, and respiratory rate. Anesthetized rats were placed on a stereotactic instrument and intracerebral injection was performed using a syringe with 10 μl volume and 0.5-mm flat needle. Lentiviral vectors (10 μl, 1 × 10^8^ TU/ml), constructed by Hanbio Biotechnology Co. (Shanghai, China), were injected into the lateral ventricles (anterioposterior = −1.2 mm; mediolateral = 1.3 mm to bregma; and dorsoventral = −3.0 mm) (25). The infusion was performed at 1 μl/min and the syringe was maintained in place for 5 min after infusion was completed. The syringe was then withdrawn gradually to prevent overflow. Coomassie Brilliant Blue (Beyotime Institute of Biotechnology, Shanghai, China) was used to confirm the injection site prior to lentivirus injection (Figure S1).

### Open Field Test

For the open field test, we used a black box with a height of 45 cm and a bottom of 40 × 40 cm, with a 4-W cold light source above the center of the box. Animal activity was recorded using Shanghai Jiliang Animal Behavior Analysis System (Shanghai, China). Behavioral tests were performed 1 week after lentivirus injection. Rats were placed in the center of the open field and their activity under light conditions was recorded by an infrared camera system and video synthesizer. Movement trajectories were analyzed by a computer analysis system. Analyzed behavioral changes included total crawling distance, number of rearings, and activity. After each experiment, the interior of the box was cleaned with 75% alcohol solution to remove the odor of the rat so that it would not affect the behavior of the next rat.

### Morris Water Maze Test

For the Morris water maze test, a water pool with a diameter of 160 cm and a height of 50 cm was used. Water depth was 31 cm, and the temperature was maintained at 23 ± 2°C. The pool was divided into four equal-sized quadrants, each with a uniquely colored pattern on the wall as a reference. A circular platform with a diameter of 9 cm and a height of 30 cm was placed in the center of the target quadrant, submerged 1 cm below the water surface. Rats were placed in the pool on the day prior to the navigation test and were allowed to swim for 2 min to acclimatize to the maze. In each trial, the rats were introduced into the maze facing the wall at one of the four designated starting points. A camera connected to a computer was placed above the maze to record swimming trajectory, total swimming distance, and time spent searching for the platform. If the rats did not find the platform within 120 s, the latency was recorded as 120 s. Following the test, these rats were placed on the platform for 15 s to allow learning and retention. Each rat was tested four times per day, each with a different starting point. There was a 30-min rest period between trials. The experiment lasted 5 days, and the average latency and total distance were recorded and analyzed using the Shanghai Jiliang Animal Behavior Analysis System. Twenty-four hours after the last place navigation test, the platform was removed and the rats were allowed to swim freely for 120 s, once for each animal. The number of target crossings in 120 s, and time and distance in target quadrant were calculated for the spatial probe test.

### Hematoxylin and Eosin Staining

The animals were anesthetized with 10% chloral hydrate after behavioral tests, and the fresh brain tissue was removed. Brains were fixed overnight by immersion in 4% paraformaldehyde. After gradient dehydration with ethanol and dimethyl benzene, the brains were embedded in paraffin. Sections 5 μm thick in coronal position at optic chiasm were made and mounted on the slides. Slides were deparaffinized and hydrated in water. They were then immersed in hematoxylin solution for 3–5 min and then rinsed in water. Following this, slides were differentiated with acid alcohol and then rinsed again. After immersing the sections in ammonia solution, they were washed in slowly running tap water. Slides were then dyed in eosin solution for 5 min, dehydrated, and sealed. Cellular morphological changes were observed under an optical microscope. Neurons were assessed as necrotic when they showed nuclear condensation, nuclear breakdown, nuclear dissolution, and cytoplasmic acidophilia. The arrangement of cells in brain regions was observed. Cell counting was manually performed in the hippocampus (subfields CA1, CA3, and DG), prefrontal cortex, and corpus striatum. We quantified three serial sections per brain region from each animal (four rats in each group) and then calculated the average.

### Quantitative Real-Time PCR (qRT-PCR)

Rats were anesthetized with chloral hydrate and decapitated. Unilateral tissue samples were taken for the molecular experiments. The hippocampus (Hip), prefrontal cortex (PFC), and corpus striatum (Str.) were removed and total RNA was extracted with the miRNeasy Mini Kit (Qiagen, Frankfurt, Germany); 1 μg of RNA was reverse transcribed using the PrimeScript RT reagent Kit (Takara, Otsu, Japan) to generate cDNA. Bulge-loop™ U6 and miR-384-5p qRT-PCR Primer Sets (one for reverse transcription and its pair for amplifying each target gene) were synthesized by RiboBio (Guangzhou, China). The forward and reverse primers for target genes were as follows: β-actin, 5′-CCTAGACTTCGAGCAAGAGA-3′ and 5′-GGAAGGAAGGCTGGAAGA-3′; DRD1, 5′-TCAAGAGGGAGACGAAAGT-3′ and 5′-ACAGAAGGGCACCATACA-3′. U6 and β-actin were used as internal controls for miR-384-5p and DRD1, respectively. The PCR experiments were run in triplicate (test–retest reliability was 0.873–0.982), and the CT value ranged between 15 and 35. The ViiA7 system (Applied Biosystems, Carlsbad, CA, USA) was used for qRT-PCR, and fold difference in transcript expression level was calculated with the 2^−ΔΔCT^ method. The fold change was calculated using the expression relative to controls.

### Western Blot

Tissues were lysed in radioimmunoprecipitation assay buffer (Beyotime Institute of Biotechnology, Shanghai, China) with protease and phosphatase inhibitors. Protein concentration was determined with a bicinchoninic acid protein assay kit (Beyotime Institute of Biotechnology). Proteins were separated on a 12% sodium dodecyl sulfate–polyacrylamide gel and transferred to a polyvinylidene difluoride membrane (Millipore, Billerica, MA, USA) that was blocked and incubated overnight at 4°C with primary antibodies against β-actin (1:3,000, 4,970 s; Cell Signaling Technology, MA, USA), DAT (1:500, sc-32258; Santa Cruz Biotechnology, Santa Cruz, CA, USA), and DRD1 (1:1,000, ab20066), CREB (1:1,000, ab32515), and phosphorylated (p-)CREB (1:5,000, ab32096) (all from Abcam, Cambridge, MA, MA, USA). The membrane was then incubated with appropriate horseradish peroxidase-conjugated secondary antibodies (1:5,000, BL003A/BL002A, Biosharp, Hefei, China). Page ruler pre-stained protein ladder (Thermo Fisher Scientific, Waltham, MA) suitable for approximate molecular weight determination was also used. Band intensity was measured using a Luminescent image analyzer AlphaView SA (Alpha Innotech Corporation, Berlin, Germany). For quantitative analysis, total proteins were used as loading controls for phosphoprotein, and the fold change was calculated using the expression in up- or down-regulation following miR-384-5p administration compared to controls.

### Luciferase Reporter Assay

The 3′ untranslated (UTR) region of the *DRD1* gene was amplified by PCR and cloned into the pmiR-RB-REPORT™ luciferase reporter vector (RiboBio). HEK293 cells were cultured in Dulbecco's Modified Eagle's Medium supplemented with 10% fetal bovine serum, 4 mM l-glutamine, and 100 mg/ml Normocin (InvivoGen, San Diego, CA, USA). The cells were co-transfected with the luciferase reporter vector containing wild-type or mutant *DRD1* 3′ UTR and miR-384-5p or control plasmid using Lipofectamine 2000 (Invitrogen, Carlsbad, CA, USA). At 48 h after transfection, relative luciferase activity was measured with the Dual Luciferase Reporter Assay Kit (Promega, Madison, WI, USA) and the GloMax luminometer (Promega). Results are expressed as the intensity ratio of Renilla to firefly luciferase. The experiment was performed in triplicate and repeated three times.

### Statistical Analysis

All data were inspected for normality to ensure their appropriateness for statistical analyses. According to the analyzed variables, Student's two-tailed *t*-test and non-parametric Mann–Whitney *U*-test were used. All data are presented as means ± standard deviation (SD) or median and interquartile range for parametric or non-parametric data, respectively. For all analyses, significance was assigned when *P* < 0.05.

## Results

### Relative Expression Level of miR-384-5p

**Figure 1:**
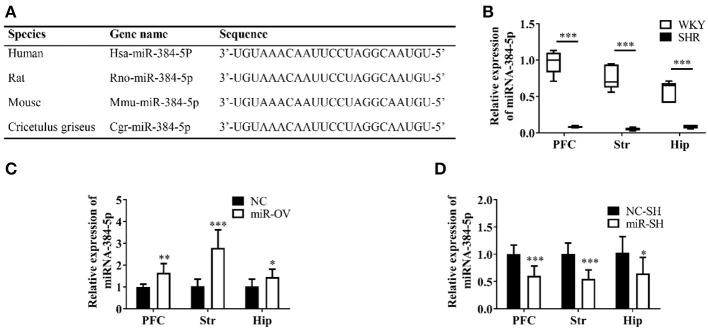
Analysis of miR-384-5p, a small non-coding RNA. **(A)** Conservation and homology of miR-384-5p in humans, mice, rats, and Chinese hamsters (*Cricetulus griseus*). **(B)** Relative expression of miR-384-5p in the PFC, Str, and Hip of WKY and SHR rats. **(C,D)** Relative expression levels of miR-384-5p that was overexpressed **(C)** or inhibited **(D)** in different brain areas, compared with control groups. *P < 0.05, **P < 0.01, ***P < 0.001.

The miR-384-5p is ubiquitously expressed in humans, rats, and mice, and has a highly conserved sequence (Figure 1A). SHR rats are one of the most frequently used animal models for investigating the behavioral dimensions of ADHD (26, 27). This is a strain that was inbred from progenitor WKY rats, which are one of the most commonly used for control groups (28). We used 4-week-old rats to evaluate the molecular basis of learning and memory deficits in ADHD. The miR-384-5p expression was lower in the brains of SHR rats [*n* = 5, median (interquartile range) values were: PFC: 0.08 (0.07, 0.10), Str: 0.59 (0.04, 0.07), Hip: 0.09 ± 0.03] compared to WKY rats [*n* = 5, PFC: 1.00 (083, 1.10), Str: 0.70 (0.62, 0.94), Hip: 0.65 (0.41, 0.69)] (*P* = 0.009; Figure 1B).

### Effects of miR-384-5p Up- or Down-Regulation

SHR rats were randomly divided into two groups that were administered with miR-384-5p overexpression vector virus (miR-OV group) or virus harboring an empty vector (NC group) by intracerebroventricular injection. The relative expression levels of miR-384-5p in the brain were evaluated by qRT-PCR. Compared to the NC group, miR-384-5p was overexpressed in the PFC (1.65 ± 0.42 *n* = 7, *P* =0.002), Str (2.79 ± 0.83, *n* = 7, *P* < 0.001), and Hip (1.46 ± 0.36, *n* = 7, *P* = 0.039; Figure 1C). At the same time, we investigated whether miR-384-5p was affected by injecting miR-384-5p-inhibitor vector (miR-SH group, *n* = 8) or empty vector control (NC-SH group, *n* = 8) delivered by lentivirus injection into the cerebroventricle of SHR rats at 4 weeks of age. We found that relative miR-384-5p expression was lower in the PFC (0.61 ± 0.18, *n* = 8, *P* < 0.001), Str (0.55 ± 0.16, *n* = 8, *P* < 0.001), and Hip (0.65 ± 0.29, *n* = 8, *P* = 0.020) of the miR-SH group compared to the control NC-SH group (Figure 1D).

### Behavioral and Histological Analyses

**Figure 2:**
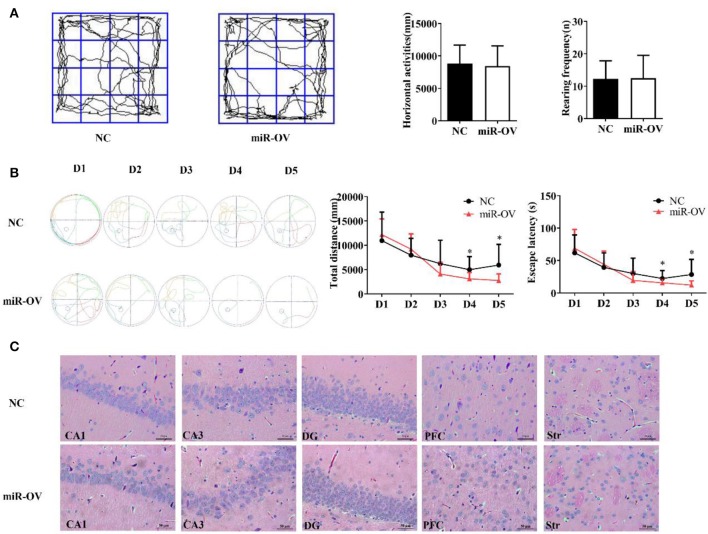
Up-regulation of miR-384-5p improves cognitive behavior without causing histological changes in brain tissue. **(A)** Path, rearing frequency, and horizontal displacement in the open field test. **(B)** Path of escape to the platform, total swimming distance, and escape latency in the Morris water maze test. *P < 0.05 **(C)** H&E staining of Hip CA1, CA3, and DG, PFC, and Str of miR-OV and NC groups. Magnification 400×.

The open field test is a standard method for assessing spontaneous activity in rats by measuring the horizontal displacement and number of rearings (29). Overexpression of miR-384-5p in the brain had no effect in terms of horizontal displacement (*P* = 0.770) and rearing frequency (*P* = 0.947; Figure 2A). In other words, spontaneous activity was similar between the two groups.

To test whether miR-384-5p expression affects spatial memory in rats, learning and memory were evaluated using the Morris water maze test (30). There were no differences in total swimming distance or escape latency in the first 3 days of training; however, time spent finding the hidden platform (escape latency) and total swimming distance were shorter in the miR-OV group than in the NC group on days 4 and 5 of the training period (*n* = 11; day 4: *P* = 0.045 and *P* = 0.019, respectively; day 5: *P* = 0.035 and *P* = 0.028, respectively; Figure 2B). In the place navigation experiment, time, and total distance traveled to reach the platform were also decreased in the miR-OV group relative to the NC group (Figure 2B). In the spatial probe test, there were no differences between the two group in the number of platform site crossings (annulus crossing, *P* = 0.904), distance traveled (*P* = 0.140), and time spent in the target platform quadrant (*P* = 0.204; Figures S2A,B). Thus, miR-384-5p overexpression improved learning and memory in SHR rats without affecting spontaneous activity.

We further investigated whether miR-384-5p overexpression induced morphological changes in the CA1, CA3, and dentate gyrus (DG) regions of the Hip, as well as in the PFC and Str 2 weeks after treatment (Figure 2C). Hematoxylin and eosin (H&E) staining revealed normal neuronal morphology in all brain regions studied of NC and miR-OV groups (*n* = 4 each), with a thick and well-organized pyramidal layer harboring high density of neurons. No obvious changes in brain structure were observed, and the number of cells was similar between groups (CA1, *P* = 0.415; CA3, *P* = 0.841; DG, *P* = 0.474; PFC, *P* = 0.690; Str., *P* = 0.725; Figure S3A).

**Figure 3:**
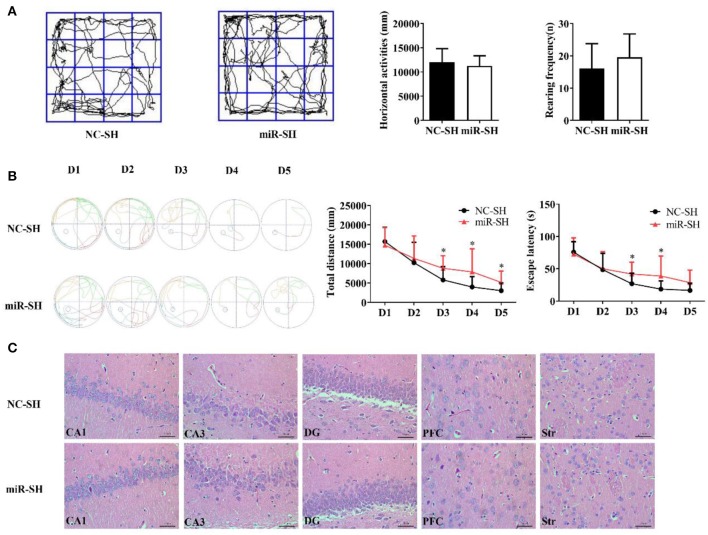
Down-regulation of miR-384-5p impairs learning and memory without causing histological changes in the brain. **(A)** Path, rearing frequency, and horizontal displacement in the open field test. **(B)** Path of escape to the platform, total swimming distance, and escape latency in the Morris water maze test. **(C)** H&E staining of Hip CA1, CA3, and DG, PFC, and Str of miR-SH and NC-SH groups. *P < 0.05.

Spatial memory following miR-384-5p suppression was also evaluated with the Morris water maze and open field tests. Horizontal displacement (*P* = 0.463) and rearing frequency (*P* = 0.263) did not differ between miR-SH and NC-SH groups (*n* = 12 each; Figure 3A). Spontaneous activities in the open field test were also similar between the two groups. In the Morris water maze test (Figure 3B), total swimming distance was higher in the miR-SH group on days 3, 4, and 5 (*P* = 0.037, 0.049, and 0.049, respectively). Furthermore, time spent searching for the platform was longer on days 3 and 4 (*P* = 0.044 and 0.048, respectively). In the spatial probe test, there were no differences in the number of annulus crossing (*P* = 0.888), distance (*P* = 0.890), and time spent in the target platform quadrant (*P* = 0.665) between the two groups (Figures S2C,D). These results suggest that suppressing miR-384-5p level in the brain negatively affects learning and memory in SHR rats. H&E staining revealed no pathological changes in the brain of either group (*n* = 4 each). Values for neuronal degeneration and reduction in the number of neurons were similar between groups in the various brain regions studied (CA1, *P* = 0.950; CA3, *P* = 0.829; DG, *P* = 0.951; PFC, *P* = 0.899; Str., *P* = 0.745; Figure 3C and Figure S3B).

### DRD1 Relative Expression

**Figure 4:**
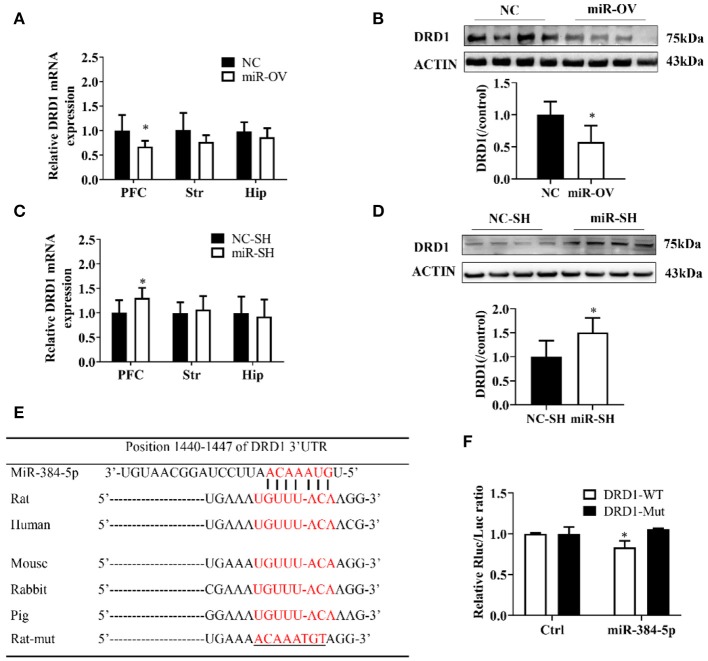
miR-384-5p up- or down-regulation alters DRD1 expression levels. **(A,B)** Relative expression levels of *DRD1* mRNA in different brain areas **(A)**, and DRD1 protein level in the PFC **(B)**, as a result of miR-384-5p overexpression. **(C,D)** Relative expression levels of *DRD1* mRNA in different brain areas **(C)** and DRD1 protein level in the PFC **(D)**, following suppression of miR-384-5p. **(E)** Complementarity between miR-384-5p seed sequence and 3′ UTR of *DRD1* mRNA, as predicted by TargetScan. **(F)** Relative luciferase activity in HEK-293T cells co-transfected with miR-384-5p or control vector and DRD1-WT or DRD1-Mut. *P < 0.05.

To clarify the molecular basis for the relationship between miR-384-5p and learning and memory in SHR rats, we used qRT-PCR and Western blot to examine the expression of DRD1, a cognition-associated receptor, in the brain, following miR-384-5p overexpression. Level of *DRD1* mRNA was lower in the miR-OV group compared to the NC group (*n* = 6 each) in the PFC (*P* = 0.041) but not in the other brain areas evaluated (Str., *P* = 0.145; Hip, *P* = 0.320; Figure 4A). DRD1 protein level in the PFC was also lower (0.58-fold) in the miR-OV group relative to control (*n* = 4 each, *P* = 0.042; Figure 4B, Figure S4A).

*DRD1* mRNA expression level in the PFC was up-regulated in the miR-SH group as compared to the NC-SH group (*n* = 6; *P* = 0.047; Figure 4C), with no changes observed in other brain regions (Str., *P* = 0.621; Hip, *P* = 0.754). DRD1 protein expression was 1.51-fold higher in the PFC of the miR-SH group (*n* = 4, *P* = 0.039; Figure 4D, Figure S4B).

A computational analysis, using TargetScan, revealed that the 3′ UTR of the *DRD1* gene contained putative miR-384-5p binding sites (Figure 4E). To clarify the relationship between miR-384-5p and *DRD1*, we examined the interaction between miR-384-5p and wild-type (DRD1-WT) or mutated (DRD1-Mut) forms of these binding sites in HEK-293T cells, using the luciferase reporter assay. We found that transfection with miR-384-5p and DRD1-WT plasmids reduced luciferase activity (i.e., lower ratio of Renilla to firefly luciferase) compared with the control (0.83 ± 0.07, *n* = 3, *P* = 0.022; Figure 4F). Transfection with DRD1-Mut abolished this effect, indicating that miR-384-5p negatively regulated *DRD1* expression.

### DAT Expression and p-CREB/CREB Ratio

**Figure 5:**
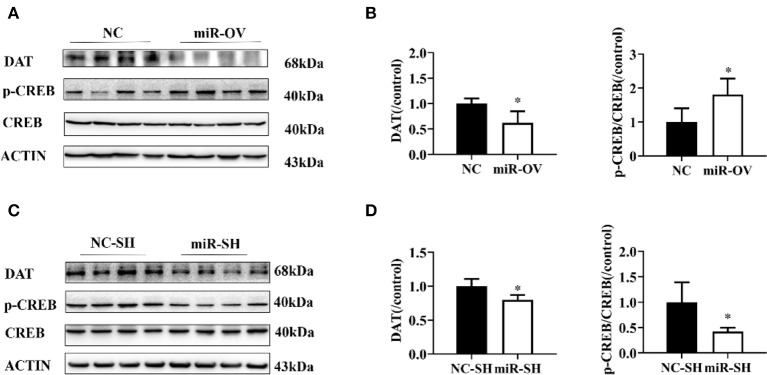
Changes in DAT protein levels and p-CREB/CREB ratio following overexpression or suppression of miR-384-5p. **(A,B)** Relative levels of DAT protein and p-CREB/CREB ratio in the PFC, following overexpression miR-384-5p. **(C,D)** DAT protein expression level and CREB/p-CREB ratio in the PFC, following suppression of miR-384-5p. *P < 0.05.

We examined whether miR-384-5p overexpression in the PFC caused changes in DAT and CREB protein expression levels and found that in comparison to the NC group, in the miR-OV group, DAT level was 0.62-fold lower (*n* = 4, *P* = 0.023) and p-CREB level was 1.80-fold higher (*n* = 4, *P* = 0.038), whereas there was no change in total CREB in the PFC (Figures 5A,B, Figure S4C). When compared to the NC-SH group, the level of DAT (0.80 folds, *P* = 0.022) and p-CREB/CREB ratio (0.42 fold, *P* = 0.028) were lower in the miR-SH group (Figures 5C,D, Figure S4D; *n* = 4 per group).

## Discussion

Many factors contribute to the etiology of ADHD, with the interaction between genetics and the environment increasing susceptibility to this disorder (2). MiRNAs modulate the expression of ADHD-associated genes, whereas genetic variants may dysregulate miRNA expression, ultimately leading to ADHD (7).

In the present study, we showed that aberrant miR-384-5p expression affected learning and memory in SHR rats. To investigate the underlying mechanisms, we examined the expression of cognition-associated genes in specific brain regions, namely, the Hip, PFC, and Str. DRD1 is one of five DA receptors expressed on the neuron surface (31). An ultrastructural analysis by electron microscopy revealed that DRD1 is distributed in the PFC and Hip and in both the pre- and postsynaptic neuronal compartments, showing that DRD1 protein is mainly present in postsynaptic dendritic spines of asymmetric synapses (32). It was reported that activation of DRD1 increases N-methyl-d-aspartate-mediated PFC pyramidal cell excitability via postsynaptic protein kinase (PK)-A activity, as well as CREB phosphorylation in rats (33). We identified DRD1 as a potential target of miR-384-5p regulation, using TargetScan (Figure 4E); this regulatory relationship was confirmed by the observation that miR-384-5p overexpression or inhibition altered DRD1 mRNA and protein levels in the PFC. The PFC is thought to be linked to the etiology of ADHD, owing to its direct and indirect connection with the Hip and Str., and its association with attention, working memory, planning, decision, and monitoring (34). ADHD was also suggested to result from defects in the prefrontal, parietal, and subcortical areas (35), while the predominant theory of ADHD is based on attentional networks involving the PFC and related areas (36). Thus, the dysregulation of miR-384-5p in SHR rats and the associated abnormalities in learning and memory might be due to aberrant DRD1 expression in the PFC.

Here, we found that the expression of DRD1 is regulated by miR-384-5p. Although miR-384-5p overexpression inhibited DRD1, it is possible that it stimulated DA release to enhance synaptic connectivity. Furthermore, optimal level of DRD1 stimulation may differ, depending on the signal-to-noise demands of a cognitive task. This is so when a task demands precise rather than broad tuning, and the subject must suppress irrelevant information (noise) for optimal performance (37). Previous studies have suggested that down-regulation of DAT expression reduces presynaptic membrane resorption of DA and increases neurite outgrowth (38, 39). CREB phosphorylation at Ser133 is induced by the adenylate cyclase/cAMP/PKA pathway via activation of DRD1 by DA (40–42). CREB plays a critical role in the central nervous system as a nexus for intracellular signaling pathways that regulate a variety of nervous system functions (43, 44). We observed that miR-384-5p down-regulation increased DRD1 protein level with a concomitant reduction in DAT and phosphorylation of CREB. It has been reported that spatial working memory is impaired in response to both excessive and insufficient DRD1 stimulation, a phenomenon known as the inverted U effect (45). High DRD1 stimulation can lead to impaired mental flexibility and affect PFC neuronal firing (37). We speculate that high DRD1 stimulation might lead to lower activation of DRD1 through regulating DA and influence CREB phosphorylation signaling pathway. DRD1 reduced presynaptic membrane DAT, possibly by regulating synaptic DA negative feedback and affecting clearance of DA. In the present study, we found that miR-384-5p overexpression alleviated the spatial learning deficits of SHR rats, whereas miR-384-5p suppression had the opposite effect, which supported the previous findings discussed above.

In conclusion, we showed that miR-384-5p possibly negatively regulates DRD1 expression. This enhances dopaminergic neurotransmission, leads to an increase in CREB phosphorylation, and improves spatial learning and memory in SHR rats. However, ADHD is a complex polygenic hereditary disorder. The interaction between multiple susceptible genes and environmental factors cannot be dismissed. Additional studies are needed to fully elucidate the role of dopaminergic neurotransmission in ADHD.

## Data Availability Statement

The datasets generated for this study are available on request to the corresponding author.

## Ethics Statement

The study protocol was approved by the Animal Ethics Committee of Nanjing Medical University (No. IACUC-1711004).

## Author Contributions

QX, JO, and QZ performed and managed the experiments, as well as performed the statistical analyses. LY and RT analyzed the data. QX wrote the first draft of the manuscript. JW and QH revised the manuscript. XG, MT, LY, and XC contributed to study design and interpretation of results. All authors read and approved the final version of the manuscript.

### Conflict of Interest

The authors declare that the research was conducted in the absence of any commercial or financial relationships that could be construed as a potential conflict of interest.

## Abbreviations

3′ UTR: 3′ untranslated region
ADHD: attention deficit hyperactivity disorder
CREB: cyclic AMP response element binding protein
DA: dopamine
DAT: dopamine transporter
DRD1: dopamine receptor D1
H&E: hematoxylin and eosin
Hip: hippocampus
miRNA: microRNA
miR-OV: microRNA overexpression
Mut: mutant
NC: negative control
p-CREB: phosphorylated cyclic AMP-response element-binding protein
PFC: prefrontal cortex
SHR: spontaneously hypertensive
Str: striatum
WT: wild type.
