# Supplementary material for: Effects of Aberrant miR-384-5p Expression on Learning and Memory in a Rat Model of Attention Deficit Hyperactivity Disorder
Source: Front Neurol. 2020 Feb 11;10:1414. doi: 10.3389/fneur.2019.01414 (PMC7026368; doi:10.3389/fneur.2019.01414)
Supplement: Supplementary file 1 [file Data_Sheet_1.pdf]

Supplementary Material

A

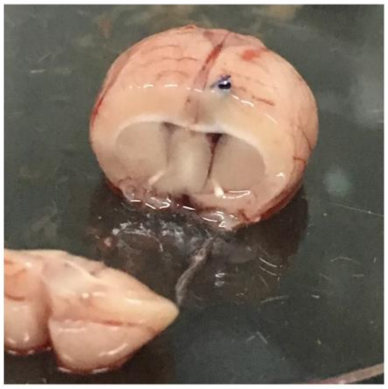

Figure S1. The injection site prior to lentivirus injection operation was confirmed from the coronal plane of brain.

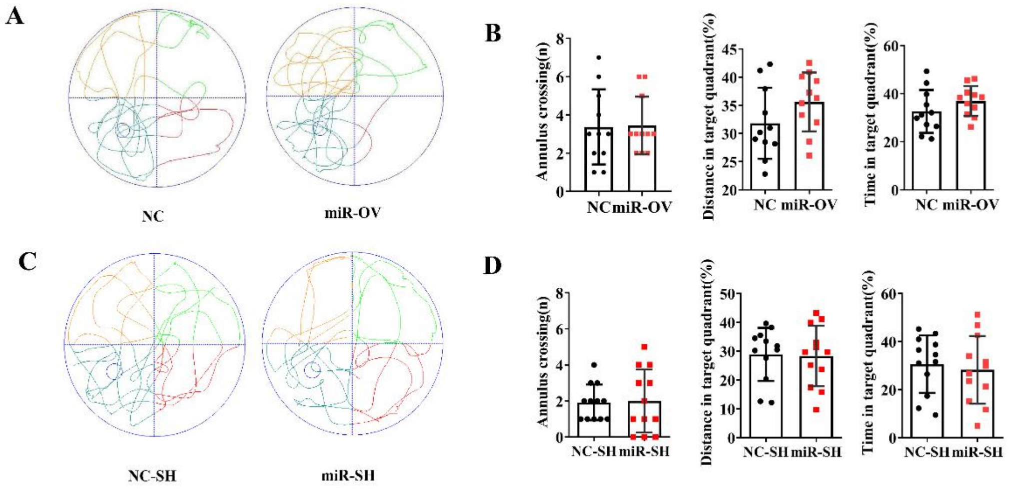

Figure S2. Up-or-down regulation of miR-384-5p make no difference on behaviors in spatial probe test. **A. C.** Representative path trace of each group in spatial probe test. **B.D.** Annulus crossing, swimming distance and time spent in target during spatial probe test.

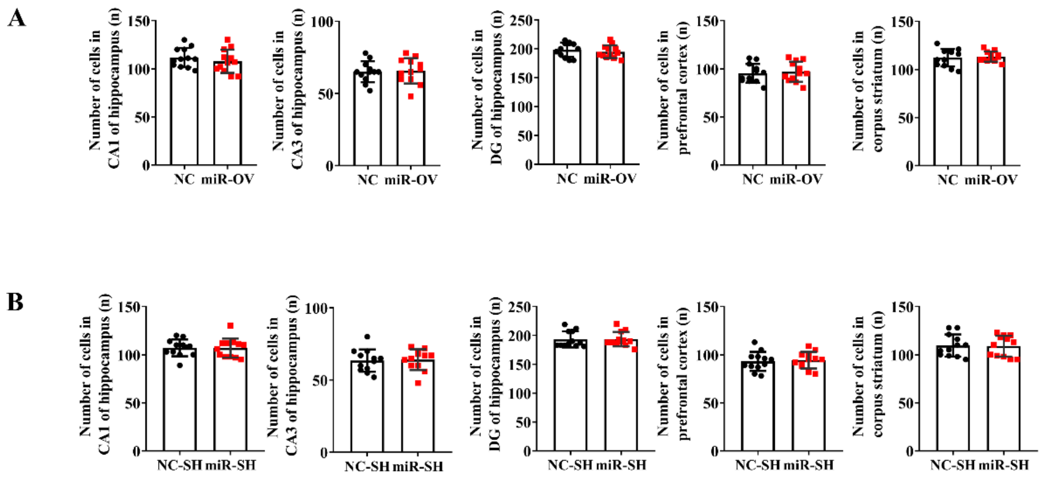

Figure S3. H&E staining revealed no pathological changes in the brain of either group such as a reduction in the number of neurons and neuronal degeneration in the Hip, PFC, and Str. **A.B.** Quantification of the numbers of cells between groups.

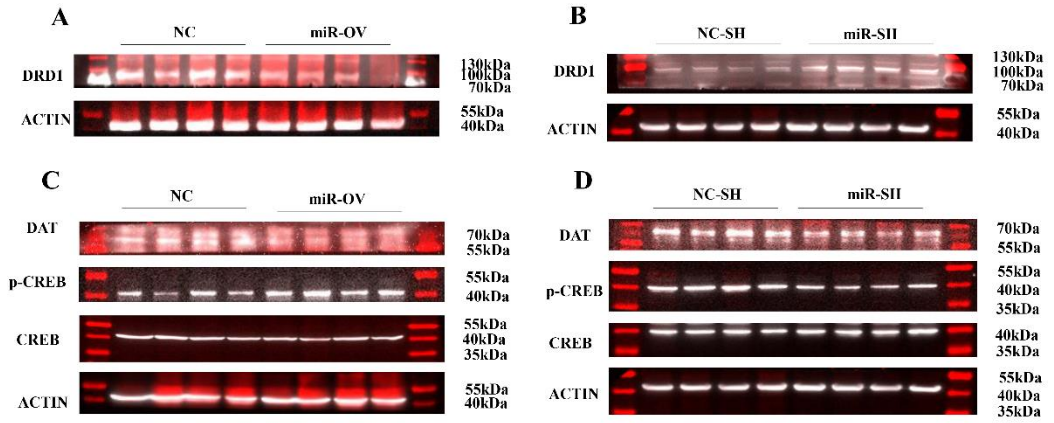

Figure S4. Changes in DRD1, DAT protein levels and p-CREB/CREB ratio following overexpression-or-suppression of miR-384-5p. **A. C.** Relative levels of DRD1, DAT protein and p-CREB/CREB ratio in the PFC, as regulation by overexpression miR-384-5p. **B.D.** DRD1, DAT protein expression and CREB/p-CREB ratio in the PFC, following suppression of miR-384-5p.
